# Signed, sealed, delivered: a generalizable model for living biotherapeutic dosing and metabolism

**DOI:** 10.1038/s41540-026-00685-4

**Published:** 2026-03-24

**Authors:** Vicenzo L. DeVito, Bhargav R. Karamched

**Affiliations:** 1 Department of Mathematics, Florida State University, Tallahassee, FL USA; 2 Institute of Molecular Biophysics, Florida State University, Tallahassee, Fl USA; 3 Program in Neuroscience, Florida State University, Tallahassee, FL USA

**Keywords:** Systems biology, Pharmacodynamics, Pharmacokinetics

## Abstract

Living Biotherapeutic Products (LBPs) offer a promising therapeutic strategy for metabolic disorders rooted in gut microbiome dysfunction, yet quantitative frameworks for predicting their efficacy remain underdeveloped. We introduce the Bacterial Compartment Absorption and Transit (BCAT) model, a pharmacokinetic-pharmacodynamic framework that couples probiotic transit, endogenous microbiome metabolism, and enzymatic transformation within a unified dose-optimization setting. Building on the classical CAT model, BCAT incorporates mechanistically-derived colon compartments and treats dosing time as a control variable. We validate BCAT against clinical data for native choline metabolism and SYNB1618 probiotic trials, achieving 5% mean prediction error compared to ~30% for prior two-compartment models. Applying BCAT to trimethylaminuria (TMAU), we predict that ~10^9^ CFU of engineered probiotic, administered 3–4 h before meals, achieves 95% reduction in systemic trimethylamine, matching healthy hepatic clearance. Global sensitivity analysis identifies enzyme expression level as the dominant design parameter, enforcing the broad applicability of this model. The BCAT framework generalizes to any gut microbiome-mediated metabolic disorder and provides quantitative dosing targets to guide live biotherapeutic development.

## Introduction

With significant advancements in genetic engineering and synthetic biology over the past twenty years, development of health technology has grown tremendously. Synthetic microbial consortia have, in particular, shown promise as delivery mechanisms for biomolecular therapy^1–6^. Engineered bacteria such as *E. coli*^7–9^, *L. lactis*^10–16^, and *B. ovatus*^10,17^ have been introduced to the gut microbiome as synthetic probiotics as a means of silencing oncogenes, inhibiting proteases, and killing harmful pathogens such as *Pseudomonas aeruginosa*^18^. Synthetic probiotics (hereafter used interchangeably with ‘probiotics’) are also being used as metabolic therapeutics, aiding expression of key biomolecules that help mitigate metabolic disorders such as diabetes^19–21^. This is particularly significant because a large percentage of metabolism incepts in the gut microbiome, which has a metabolic capacity containing over three million genes—larger than that of the liver^18,22–25^.

As synthetic probiotics develop and become mainstream in biomolecular therapy, quantitative tools that inform and optimize therapeutics will become increasingly important for them. Indeed, integration of experimental and theoretical techniques has been successful in accelerating understanding of, for example, the role biomolecular oscillations play in coordinating activity across engineered microbial consortia, especially in spatially extended domains^2,26–30^. Mathematical modeling has not been as integrated in biomolecular therapy of the gut microbiome. In particular, only a few mathematical models have been developed that incorporate transfer of probiotic through the gastrointestinal (GI) tract as it performs a desired metabolic function.

In this paper, we present the first compartmental pharmacokinetic-pharmacodynamic (PK-PD) framework that explicitly couples probiotic transit, endogenous microbiome metabolism, and enzymatic transformation within a unified dose-optimization setting. Our model also optimally incorporates compartments for the colon, which has largely been ignored as a source of metabolism until recently. Our model is based on the compartment absorption and transit (CAT) model prevalent in physiologically-based pharmacokinetic (PBPK) modeling^31–33^.

The CAT model treats the small intestine as a one-dimensional spatial domain, tracking the dynamics of ingested substances as they transit from stomach to colon. Mathematically, the model discretizes an advection-absorption partial differential equation. If *ρ*(*x*, *t*) denotes the concentration of a substance at position *x* ∈ (0, *L*) at time *t* > 0, then

1 $$\frac{\partial \rho }{\partial t}=-v\frac{\partial \rho }{\partial x}-{K}_{a\rho }\rho ,$$

where *v* is the transit velocity and *K*_*aρ*_ is the absorption rate across the intestinal wall. An upwind finite-difference discretization^34,35^ yields

2 $$\frac{d{\rho }_{i}}{dt}=-v\frac{{\rho }_{i}-{\rho }_{i-1}}{\Delta x}-{K}_{a\rho }{\rho }_{i}={K}_{t}({\rho }_{i-1}-{\rho }_{i})-{K}_{a\rho }{\rho }_{i},$$

where *ρ*_*i*_(*t*) is the concentration in compartment *i* (*i* = 1, …, *N*), *Δx* is the length of each compartment, and *K*_*t*_ ≡ *v*/*Δx* is the transit rate constant—equivalently, the inverse of the residence time within each compartment. For the first compartment, gastric emptying at rate *K*_*e*_ replaces the transit inflow:

3 $$\frac{d{\rho }_{1}}{dt}={K}_{e}{\rho }_{s}-{K}_{t}{\rho }_{1}-{K}_{a\rho }{\rho }_{1}$$

The model is closed by incorporating equations for the stomach (*ρ*_s_), colon (*ρ*_c_), and plasma (*ρ*_pl_):

4 $$\frac{d{\rho }_{s}}{dt}=-{K}_{e}{\rho }_{s}$$

5 $$\frac{d{\rho }_{c}}{dt}={K}_{t}{\rho }_{N}$$

6 $$\frac{d{\rho }_{{\mathrm{pl}}}}{dt}={K}_{a\rho }\mathop{\sum }\limits_{i=1}^{N}{\rho }_{i}-{K}_{{\rm{e}}{\rm{l}}}{\rho }_{{\mathrm{pl}}}$$

where *K*_*e*_ is the gastric emptying rate and *K*_el_ is the plasma elimination rate.

The CAT model assumes that transit velocity is constant along the intestine and that absorption is homogeneous across all compartments. Using *N* = 7 compartments optimally captures human small intestinal transit time distributions^31,36,37^. Despite its simplicity, the CAT model has proven highly successful and forms the foundation of the ACAT framework implemented in GastroPlus™^38,39^, the industry standard for oral drug absorption modeling.

Several mechanistic frameworks have extended compartmental modeling to engineered living therapeutics. Charbonneau et al. introduced a two-compartment model for SYNB1618 (phenylalanine degradation) that captured essential dose-response relationships^40^. Lubkowicz et al. extended this with a three-compartment ODE model for SYNB8802 (oxalate degradation), incorporating a colonic sink^41^. The ALT-CAT model of Mays and Nair adapts the ACAT framework with seven small intestinal compartments and a “Therapeutic Factor” metric integrating bacterial population, enzyme activity, and substrate transport^42^.

Our framework addresses several limitations of these approaches. First, we model probiotic populations as dynamic entities transiting through the GI tract rather than static distributions. Second, we incorporate mechanistically-derived colon compartments by fitting to radiopaque marker transit data. Third, whereas previous models focused on substrate detoxification, BCAT captures competitive interactions in which the probiotic intercepts a harmful intermediate produced by the endogenous microbiome, requiring explicit modeling of resident gut bacterial metabolism. Finally, we treat dosing time as a control variable, enabling joint optimization of dose and timing. We validate BCAT against both native microbiome metabolism and SYNB1618 clinical data, achieving substantially improved predictive accuracy over prior approaches. For concreteness, we apply our BCAT model to trimethylaminuria.

Trimethylaminuria (TMAU) is a metabolic disorder characterized by the accumulation of trimethylamine (TMA), a volatile compound with a pungent fishy odor, in bodily fluids^43^. The condition arises from mutations in the *FMO3* gene encoding flavin-containing monooxygenase 3 (FMO3), which normally oxidizes > 95% of absorbed TMA to odorless trimethylamine *N*-oxide (TMAO)^44^.

TMA enters the bloodstream primarily through gut bacterial metabolism of dietary precursors: choline, betaine, and L-carnitine, via enzyme complexes including CutC/CutD, CntA/B, and yeaW/X^45–47^. Direct dietary intake of TMA (e.g., from seafood) contributes a smaller fraction^48^. Following intestinal absorption, TMA is transported to the liver via the portal vein, where FMO3-mediated oxidation normally prevents systemic accumulation.

TMAU affects an estimated 1 in 200,000 to 1 in 1,000,000 individuals worldwide^49,50^. No cure exists; current management relies on dietary restriction of TMA precursors, antibiotics to suppress gut bacteria, and activated charcoal—all of which address symptoms rather than mechanism^43,51^.

Emerging approaches target the bacterial enzymes responsible for TMA production. Small-molecule inhibitors of CutC/CutD, such as iodomethylcholine (IMC) and fluoromethylcholine (FMC), achieve > 90% reduction in plasma TMAO in preclinical models^52^. However, no human trials have been conducted, and concerns remain regarding microbiome disruption, off-target effects on host choline metabolism, and incomplete pathway coverage—CntA/B, yeaW/X, and betaine reductase continue producing TMA from non-choline precursors^47,53,54^.

Live biotherapeutic products (LBPs) offer an alternative strategy: rather than inhibiting TMA production, an engineered probiotic expressing trimethylamine monooxygenase (TMM)—the bacterial homolog of FMO3^55^—could oxidize TMA directly within the gut lumen before absorption. This approach would degrade TMA regardless of its precursor pathway, without interfering with host choline biology.

## Results

**Fig. 1:**
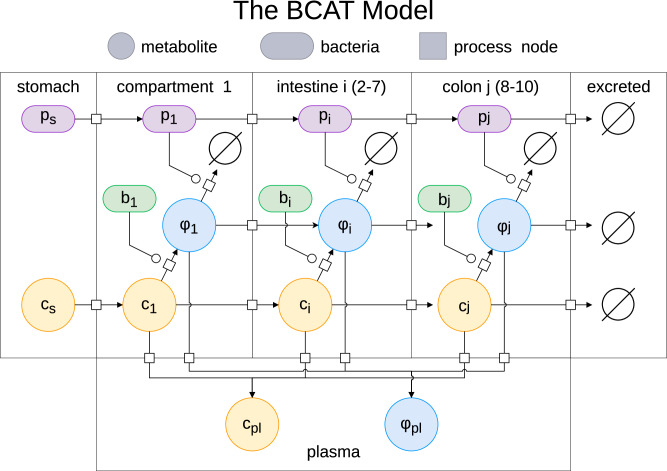
The BCAT model. Schematic of the BCAT model.

Here we present dosage predictions for probiotic to treat TMAU. For details on the BCAT model itself and the definitions of various parameters, please see Methods and Fig. 1.

### Simulating trimethylaminuria

We define therapeutic efficacy through the ratio of cumulative TMA absorption with and without probiotic treatment. Let

7 $${J}_{\varphi }(t)\equiv {K}_{a\varphi }\mathop{\sum }\limits_{i=1}^{7}{\varphi }_{i}+{K}_{a\varphi ,\mathrm{co}}\mathop{\sum }\limits_{j=1}^{3}{\varphi }_{\mathrm{co},j}$$

denote the instantaneous physicochemical flux of TMA across the intestinal lining, where the sum is over all gut compartments (small intestine and colon). The therapeutic ratio is

8 $${\Gamma }^{* }\equiv {\rm{l}}{\rm{i}}{{\rm{m}}}_{t\to \infty }\frac{{\int }_{0}^{t}{J}_{\varphi }^{{\mathrm{pr}}}(s)\,ds}{{\int }_{0}^{t}{J}_{\varphi }^{{\mathrm{npr}}}(s)\,ds}$$

where superscripts ‘pr’ and ‘npr’ denote presence and absence of probiotic, respectively. Our objective is to determine the minimum probiotic dose *p*_0_ achieving Γ^*^ = 0.05—i.e., 95% reduction in systemic TMA exposure, replicating the oxidative capacity of hepatic FMO3 in healthy individuals^56^. In the formalism of the BCAT model, we seek the optimal dosage *p*_0_ such that 100(1 − Γ^*^) = 95.

#### Baseline: no treatment

**Fig. 2:**
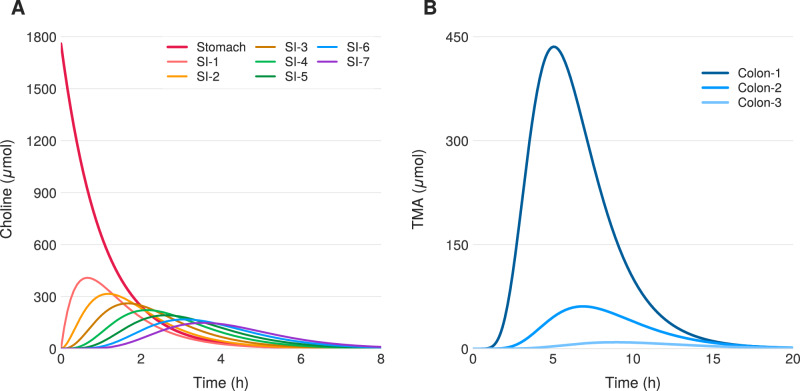
Simulation of the no treatment case. Temporal dynamics of choline and TMA across gastrointestinal compartments following a 1760 *μ*mol choline bolus. **A** Choline transit through stomach and small intestine. **B** TMA production dominated by proximal colon, peaking at ~440 *μ*mol around 5 hours.

In the baseline simulation, probiotic is not administered (*p*_0_ = 0), leading to unrestrained conversion of choline into TMA and subsequent absorption into the portal vein. Figure 2 shows time series of choline and TMA concentrations across intestinal compartments. The solutions resemble those of a linear transport equation with decay—a traveling wave with decay constant determined by absorption across the intestinal lining. Choline enters the small intestine from the stomach, yielding exponential decay in compartment 1. Throughout subsequent compartments, choline concentration in compartment *i* rises toward that of compartment *i* − 1, then falls due to absorption and bacterial metabolism. Dynamics in compartments 1–7 of the small intestine and 1–3 of the colon show the emergence of TMA as a metabolic product of choline. The TMA dynamics also resemble a peaked response in all compartments.

#### Analytical result

When *c*_0_ ≪ *K*_*mb*_ and *φ* ≪ *K*_*m*_, both bacterial conversion and probiotic degradation are well-approximated by a first-order Taylor series:

9 $$g(b,c)\approx {K}_{bc}\,b\,c,\,f(p,\varphi )\approx {K}_{p}\,p\,\varphi$$

where $${K}_{bc}\equiv {{\mathcal{V}}}_{\max }/{K}_{mb}$$ and *K*_*p*_ ≡ *k*_cat_*E*_eq_/*K*_*m*_.

*Baseline* (*p*_0_ = 0) The linearized system admits closed-form solutions via convolution integrals. Define the effective decay rates:

$$\begin{array}{clcclc}{\lambda }_{i} & \equiv {K}_{ac}+{K}_{t}+{K}_{bc}{b}_{i} & ({\rm{S}}{\rm{I}}) & {\lambda }_{{\rm{c}}{\rm{o}},j} & \equiv {K}_{ct}+{K}_{bc}{b}_{{\rm{c}}{\rm{o}},j} & ({\mathrm{colon}})\\ \mu & \equiv {K}_{a\varphi }+{K}_{t} & ({\rm{S}}{\rm{I}}) & {\mu }_{{\rm{c}}{\rm{o}}} & \equiv {K}_{a\varphi,co }+{K}_{ct} & ({\mathrm{colon}})\end{array}$$

The time-integrated choline concentrations are:

10 $${C}_{i}=\frac{{K}_{t}^{i-1}{c}_{0}}{{\prod }_{j=1}^{i}{\lambda }_{j}}\,\,\,(i=1,\ldots ,7)$$

11 $${C}_{{\rm{c}}{\rm{o}},j}=\frac{{K}_{t}^{7}{K}_{ct}^{j-1}{c}_{0}}{{\prod }_{k=1}^{7}{\lambda }_{k}{\prod }_{m=1}^{j}{\lambda }_{{\rm{c}}{\rm{o}},m}}\,\,\,(j=1,2,3)$$

The time-integrated TMA concentrations are:

12 $${\Phi }_{i}^{({\rm{b}}{\rm{a}}{\rm{s}}{\rm{e}})}=\frac{{K}_{bc}}{\mu }\mathop{\sum }\limits_{k=1}^{i}{b}_{k}{C}_{k}{(\frac{{K}_{t}}{\mu })}^{i-k}\,\,\,(i=1,\ldots ,7)$$

13 $${\Phi }_{{\rm{c}}{\rm{o}},j}^{({\rm{b}}{\rm{a}}{\rm{s}}{\rm{e}})}=\frac{{K}_{t}{\Phi }_{7}^{({\rm{b}}{\rm{a}}{\rm{s}}{\rm{e}})}}{{\mu }_{{\rm{c}}{\rm{o}}}}{\left(\frac{{K}_{ct}}{{\mu }_{\mathrm{co}}}\right)}^{j-1}+\frac{{K}_{bc}}{{\mu }_{{\rm{c}}{\rm{o}}}}\mathop{\sum }\limits_{m=1}^{j}{b}_{{\rm{c}}{\rm{o}},m}{C}_{{\rm{c}}{\rm{o}},m}{\left(\frac{{K}_{ct}}{{\mu }_{\mathrm{co}}}\right)}^{j-m}\,\,\,(j=1,2,3)$$

The cumulative TMA flux into plasma is:

14 $${\int }_{0}^{\infty }{J}_{\varphi }^{({\rm{b}}{\rm{a}}{\rm{s}}{\rm{e}})}dt={K}_{a\varphi }\mathop{\sum }\limits_{i=1}^{7}{\Phi }_{i}^{(\mathrm{base})}+{K}_{a\varphi ,\mathrm{co}}\mathop{\sum }\limits_{j=1}^{3}{\Phi }_{\mathrm{co},j}^{(\mathrm{base})}$$

These equations reveal which biological parameters control TMA absorption in the absence of probiotic. Reducing *K*_*aφ*_ → 0 (TMA absorption rate) clearly reduces systemic TMA. Other strategies suggested by the analytic solution include:

1. Eliminate choline consumption (*c*_0_ → 0)
2. Prevent bacterial metabolism (*K*_*bc*_ → 0)
3. Eliminate gut bacteria (*b*_*k*_ → 0)
4. Increase choline absorption (*K*_*ac*_ → *∞*), reducing substrate available for bacterial conversion
5. Increase intestinal transit (*K*_*t*_ → *∞*), reducing residence time for bacterial metabolism

Current TMAU management addresses strategies (1) and (3): dietary choline restriction and antibiotics to suppress gut bacteria. However, these approaches are nutritionally restrictive and unsustainable for long-term management. Strategies (4) and (5) are physiologically impractical with no clear implementation path. This motivates the probiotic approach: rather than eliminating TMA production, we intercept TMA before absorption.

### Probiotic treatment

We now take *p*_0_ > 0 and generate a dose-response curve to determine the minimum dose yielding Γ^*^ = 0.05. For each value of *p*_0_, we solve the BCAT model to equilibrium and plot the resulting equilibrium ratio Γ^*^ against *p*_0_. The minimal effective dose is the smallest *p*_0_ at which Γ^*^≤0.05. We assume the probiotic is co-administered with the meal unless otherwise stated.

**Fig. 3:**
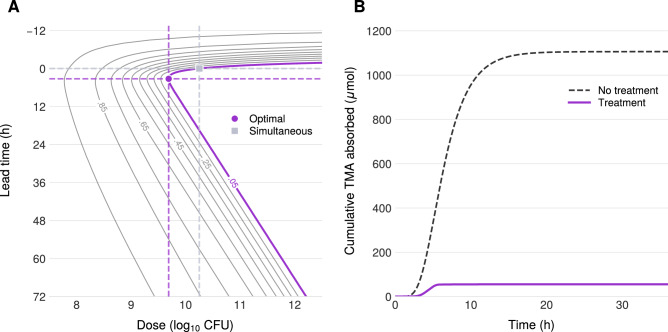
Simulated dose-time response. Results of treatment. **A** Dose response curve describing TMA flux into the plasma as a function of both probiotic dose and lead time. The distinct curves are indexed by the achieved Γ^*^ value. **B** Sample time series for net effect of probiotic intervention upon TMA flux. Here, Γ^*^ = 0.05.

Figure 3A, B shows the impact of probiotic on TMA flux into plasma. At the dose achieving Γ^*^ = 0.05, accumulation of TMA into the plasma decreases by approximately a factor of 20 relative to baseline. This reduction in systemic TMA corresponds directly to reduced malodor in affected individuals.

The dose response is characterized in Fig. 2A as a function of *p*_0_ and lead time (discussed in Earlier probiotic administration below). The dose-response for simultaneous ingestion can be read by following the horizontal line at lead time = 0 in Fig. 2A. The model predicts that a dose of approximately 10^10^ CFU achieves Γ^*^ = 0.05 (grey square). This dose is consistent with typical probiotic formulations^57^; approximately 10^10^ CFU fits in a standard over-the-counter supplement capsule. This dose achieves Γ^*^ = 0.05, coinciding with the TMA clearance efficiency observed in healthy individuals with functional FMO3.

The probiotic intervention thus achieves two therapeutic goals:

1. Reduced systemic TMA, diminishing the characteristic malodor
2. Restoration of 95% TMA clearance efficiency, matching healthy FMO3 function

#### Analytical result

We again assume *c*_0_ ≪ *K*_*mb*_ and *φ* ≪ *K*_*m*_, so that both bacterial conversion and probiotic degradation can be well-approximated by first-order Taylor polynomial:

15 $$\begin{array}{cc}g(b,c)\approx {K}_{bc}\,b\,c,\,\, & f(p,\varphi )\approx {K}_{p}\,p\,\varphi \end{array}$$

where $${K}_{bc}\equiv {{\mathcal{V}}}_{\max }/{K}_{mb}$$ and *K*_*p*_ ≡ *k*_cat_*E*_eq_/*K*_*m*_.

The probiotic cascade (assuming *K*_*e*_ = *K*_*t*_) yields gamma-distributed concentrations with cumulative exposure:

16 $${P}_{i}(t)=\frac{{p}_{0}}{{K}_{t}}\left[1-{e}^{-{K}_{t}t}\mathop{\sum }\limits_{m=0}^{i}\frac{{({K}_{t}t)}^{m}}{m!}\right]$$

With probiotic, the TMA solution via variation of parameters is:

17 $${\varphi }_{i}(t)={\int }_{0}^{t}{S}_{i}(\tau )\exp (-\mu (t-\tau )-{K}_{p}[{P}_{i}(t)-{P}_{i}(\tau )])d\tau$$

where *S*_*i*_(*t*) = *K*_*bc*_*b*_*i*_*c*_*i*_(*t*) + *K*_*t*_*φ*_*i*−1_(*t*).

Taylor expanding the probiotic attenuation gives:

18 $${\Phi }_{i}^{(treat)}=\mathop{\sum }\limits_{n=0}^{\infty }\frac{{(-{K}_{p})}^{n}}{n!}{I}_{i}^{(n)}$$

For compartment 1, the integral factors as ${I}_{1}^{(n)}={p}_{0}^{n}{J}_{1,n}{M}_{n}$ where:

19 $${J}_{1,n}=\frac{{K}_{bc}{b}_{1}{K}_{e}{c}_{0}}{({K}_{e}+n{K}_{t})({\lambda }_{1}+n{K}_{t})}$$

20 $${M}_{n}=\mathop{\sum }\limits_{k=0}^{n}\left(\begin{array}{l}n\\ k\end{array}\right)\frac{{(-1)}^{k}}{\mu +k{K}_{t}}$$

Higher compartments and colon follow the same structure with elementary integrals.

#### Therapeutic ratio

The therapeutic ratio, Γ^*^, can be represented in terms of *Φ*^(treat)^ and *Φ*^(base)^, yielding

21 $${\Gamma }^{* }=\frac{{\sum }_{k}{\Phi }_{k}^{({\mathrm{treat}})}}{{\sum }_{k}{\Phi }_{k}^{({\mathrm{base}})}}=1-{K}_{p}\frac{{\sum }_{k}{I}_{k}^{(1)}}{{\sum }_{k}{\Phi }_{k}^{({\rm{b}}{\rm{a}}{\rm{s}}{\rm{e}})}}+O({K}_{p}^{2})$$

For constant probiotic concentration $\overline{p}$ across all compartments,

22 $${\Gamma }^{* }\approx \frac{\mu }{\mu +{K}_{p}\overline{p}}$$

Setting Γ^*^ = 0.05 yields $$\overline{p}=19\mu /{K}_{p}$$.

#### Earlier probiotic administration

The BCAT model enables optimization of probiotic dosing time relative to meal consumption. Let *τ* denote the lead time: if choline is ingested at time *T*, probiotic is administered at time *T* − *τ*. Here, $\tau \in {\mathbb{R}}$: *τ* > 0 indicates probiotic ingestion before choline consumption, and *τ* < 0 indicates probiotic ingestion after.

Figure 2A shows dose response in the (*p*_0_, *τ*) parameter space. Each curve is a level set of constant Γ^*^, generated by systematically varying *τ* and *p*_0_ and solving the BCAT model to equilibrium.

The model predicts that earlier probiotic administration substantially reduces the dose required to achieve Γ^*^ = 0.05. Specifically, administering probiotic approximately 3–4 h before choline consumption reduces the required dose by approximately 4-fold compared to simultaneous ingestion (purple disk vs. gray square in Fig. 2A). This finding has practical significance: minimizing probiotic dose may reduce potential risks associated with high bacterial loads, including antibiotic resistance gene transfer, which poses growing concern for antimicrobial treatment efficacy^58,59^.

The mechanistic basis for this effect lies in the transit dynamics. A priori, endogenous gut bacteria are resident throughout the GI tract, with the highest densities in the colon where over 99% of TMA production occurs. Orally administered probiotic, in contrast, must transit through the stomach and small intestine before reaching the colon. While probiotic flows through the GI tract at the same rate as choline (*K*_*t*_ in the small intestine, *K*_*ct*_ in the colon), with simultaneous ingestion the delay between choline arrival, bacterial conversion to TMA, and probiotic-mediated oxidation allows substantial TMA absorption before the probiotic can act. Higher doses compensate for this temporal mismatch.

Earlier probiotic administration allows the therapeutic bacteria to reach the colon and distribute across compartments before the choline bolus arrives. When TMA production begins, probiotic is already positioned to intercept it, achieving Γ^*^ = 0.05 with lower doses.

However, administering probiotic too early is counterproductive: the probiotic transits through and exits the colon before choline arrives, diminishing its presence when needed. The optimal lead time balances these opposing effects—early enough for probiotic to establish colonic presence, but not so early that it is excreted before the substrate arrives.

### Global sensitivity analysis

To identify which parameters most strongly influence therapeutic efficacy, we performed variance-based global sensitivity analysis using Sobol’ indices^60–63^. Unlike local sensitivity methods, Sobol’ analysis quantifies parameter influence across the entire feasible parameter space, accounting for nonlinear effects and parameter interactions.

**Fig. 4:**
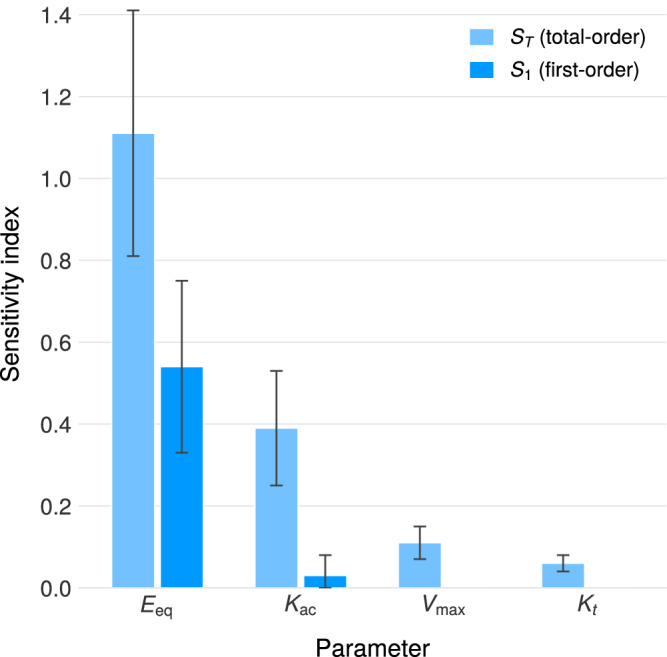
Global sensitivity analysis. Sobol sensitivity indices for the BCAT model with probiotic intervention. Enzyme expression level (*E*_eq_) dominates therapeutic outcome, with substantial parameter interactions indicated by the difference between total-order (*S*_*T*_) and first-order (*S*_1_) indices.

The model output is dominated by enzyme expression level (*E*_eq_), with ${S}_{1,{E}_{eq}}=0.54\pm 0.21$ and ${S}_{T,{E}_{eq}}=1.11\pm 0.30$. The total-order index exceeds unity, reflecting estimation uncertainty at the sample size used; the substantial interaction effect nonetheless indicates strong coupling with other parameters (see Fig. 4), primarily with choline absorption rate (${S}_{T,{K}_{ac}}=0.39\pm 0.14$, interaction effect *S*_*T*_ − *S*_1_ = 0.36).

Secondary parameters showing moderate influence include:

- $${S}_{T,{V}_{max,TMM}}=0.11\pm 0.04$$ (TMM kinetics)
- $${S}_{T,{K}_{t}}=0.06\pm 0.02$$ (intestinal transit)
- $${S}_{T,{K}_{a\varphi }}=0.03\pm 0.01$$ (TMA absorption)

All other parameters have negligible sensitivity indices (*S*_*T*_ < 0.02): bacterial kinetics (*k*_bact_, *B*_CO_, *B*_SI_), gastric emptying (*K*_*e*_), and colonic TMA absorption fraction (*f*_co_).

The dominance of *E*_eq_ persists across varying choline doses (*c*_0_ = 0.8–3.2 mmol) and probiotic doses (*p*_0_ = 10^9^–10^11^ CFU).

#### Interpretation

The dominance of *E*_eq_ has important implications for LBP development:

1. **Engineering priority:** Enzyme expression level per cell is the critical design variable. Efforts to improve therapeutic efficacy should focus on maximizing *E*_eq_ through promoter engineering, gene copy number optimization, or protein stability enhancements.
2. **Robustness to physiological variability:** Transit rates (*K*_*t*_, *K*_*ct*_, *K*_*e*_) and absorption rates (*K*_*ac*_, *K*_*aφ*_) contribute minimally to output variance. This suggests the model predictions are robust to inter-individual variability in GI physiology, supporting generalizability across patient populations.
3. **Reduced measurement burden:** Precise characterization of host physiological parameters is not critical for dose prediction. Clinical translation can proceed without patient-specific GI transit measurements.
4. **Strain selection criteria:** When selecting or engineering probiotic strains for TMA oxidation, high TMM expression level should be prioritized over optimization of other kinetic parameters. Strains with demonstrated high-level recombinant protein expression are preferred chassis organisms.

The substantial interaction between *E*_eq_ and *K*_*ac*_ (${S}_{T,{E}_{eq}}-{S}_{1,{E}_{eq}}=0.57$) indicates that the therapeutic effect depends synergistically on both enzyme expression and choline absorption dynamics. This interaction is mechanistically sensible: higher choline absorption leaves less substrate for bacterial TMA production, while higher enzyme expression increases TMA degradation capacity. All other parameters show negligible interactions (*S*_*T*,*i*_ − *S*_1,*i*_ < 0.1), indicating approximately additive behavior.

## Discussion

We developed the Bacterial Compartmental Absorption and Transit (BCAT) model, a pharmacokinetic framework for predicting live biotherapeutic efficacy in metabolic disorders involving gut microbiome dysfunction. Building on the classical CAT model—which we showed arises from upwind discretization of an advection-absorption PDE—BCAT incorporates three key extensions: (1) explicit colon compartments fitted to radiopaque marker transit data, (2) endogenous bacterial metabolism of dietary substrates, and (3) dynamic probiotic transit with Michaelis-Menten enzyme kinetics.

Several ACAT-derived frameworks have previously modeled engineered living therapeutics, most notably the ALT-CAT model of Mays and Nair^42^, which treats administered microbes as enzyme delivery vehicles for substrate detoxification—an important advance in integrating probiotic transit with host pharmacokinetics. BCAT addresses a distinct biological scenario: competitive interception of a harmful intermediate (TMA) produced by the endogenous microbiome, rather than direct detoxification of a dietary substrate. This necessitates explicit modeling of resident gut bacterial metabolism alongside probiotic activity, as well as consideration of nonlinear competition for shared substrates and intermediates. Moreover, BCAT treats dosing time as a control variable, enabling joint optimization of dose and timing—a capability absent from prior frameworks. These features extend beyond payload-centric detoxification models and position BCAT as a general PK-PD framework for studying competitive microbiome-host-probiotic interactions in systems where harmful and protective metabolites coexist.

To our knowledge, BCAT is the first compartmental PK-PD framework that explicitly couples probiotic transit, endogenous microbiome metabolism, and enzymatic transformation within a unified dose-optimization setting. The model is computationally efficient, admits closed-form solutions in the linear regime, and generalizes readily to alternative enzymatic pathways beyond the Michaelis-Menten kinetics employed here. The closed-form solutions reveal explicit parameter dependencies, enabling rapid exploration of intervention strategies without numerical simulation.

We validated BCAT against two independent datasets. First, simulations of native microbiome choline metabolism reproduced urinary TMA/TMAO excretion data from three clinical studies spanning five decades (1951–1999), confirming consistent 63–65% bacterial conversion efficiency. Second, application to SYNB1618 clinical trial data achieved 5% mean prediction error, compared to approximately 30% for prior two-compartment models. A key mechanistic insight emerged: sensitivity analysis revealed that 99.8% of TMA production occurs in the colon rather than the small intestine, validating the necessity of explicit colon compartments.

Applying BCAT to trimethylaminuria (TMAU), we predict that approximately 10^9.7^ CFU of TMM-expressing probiotic, administered 3–4 hours before a choline-rich meal, achieves Γ^*^ = 0.05—i.e., 95% reduction in systemic TMA exposure, matching the clearance efficiency of healthy hepatic FMO3. Simultaneous administration requires higher doses but remains within clinically achievable ranges. Global sensitivity analysis identified enzyme expression level (*E*_eq_) as the dominant parameter controlling therapeutic outcome, suggesting that strain engineering efforts should prioritize maximizing per-cell enzyme activity. The robustness of model predictions to physiological parameter variation (demonstrated via Sobol’ analysis) supports generalizability across patient populations, facilitating clinical translation without patient-specific GI transit measurements.

Several limitations warrant acknowledgment. The model assumes passive probiotic transit without growth, death, or colonization—appropriate for single-dose pharmacokinetics but potentially limiting for repeated dosing scenarios. Bacterial density profiles were approximated from literature ranges rather than patient-specific measurements. Intracellular enzyme dynamics and membrane transport were not explicitly modeled. Additionally, no TMM-expressing probiotics have been clinically tested; our predictions await experimental validation.

Future work will address these limitations through three avenues. First, incorporating bacterial population dynamics (growth, death, competitive exclusion) would extend BCAT to chronic dosing regimens. Second, patient-specific parameterization using microbiome profiling could improve individual predictions. Third, and most directly, we are pursuing experimental validation through synthesis of TMM-expressing *B. subtilis* strains and in vitro characterization of TMA oxidation kinetics. The BCAT framework provides quantitative dosing targets to guide this synthetic biology effort and, more broadly, offers a generalizable platform for modeling competitive microbiome-probiotic interactions across metabolic disorders.

## Methods

In this section, we discuss details of how we constructed and validated the BCAT model. We also discuss fundamentals of Sobol’ global sensitivity analysis.

### Extending BCAT to the colon

Given that TMA production predominantly occurs in the colon, accurately modeling LBP efficacy requires explicit colon compartments—yet the classical CAT framework terminates at the ileocecal junction. We extend the model in two steps: first fitting colonic transit dynamics, then incorporating bacterial metabolism.

We add *N* colon compartments following the fitting procedure of Lawrence et al.^31^. For an inert, non-absorbed marker, the fraction of dose in each colon compartment evolves as:

23 $$\frac{d{f}_{{\mathrm{co}},1}}{dt}={K}_{t}{f}_{7}-{K}_{ct}{f}_{{\mathrm{co}},1}$$

24 $$\frac{d{f}_{{\mathrm{co}},i}}{dt}={K}_{ct}({f}_{{\mathrm{co}},i-1}-{f}_{{\mathrm{co}},i}),\,i=2,\ldots ,N$$

25 $$\frac{d{f}_{{\mathrm{exc}}}}{dt}={K}_{ct}{f}_{{\mathrm{co}},N}$$

where *f*_co,*i*_ is the fraction of dose in the *i*th colon compartment, *f*_exc_ is the cumulative excreted fraction, and *K*_*ct*_ = *N*/〈*T*_co_〉 is the colonic transit rate.

We obtained marker excretion data from Metcalf et al.^64^, who measured radiopaque marker transit in healthy subjects, reporting mean colonic transit time 〈*T*_co_〉 = 35 h. Radiopaque markers are ideal for transit fitting: inert, non-absorbed, and non-degraded, they track bulk flow without metabolic confounds.

**Fig. 5:**
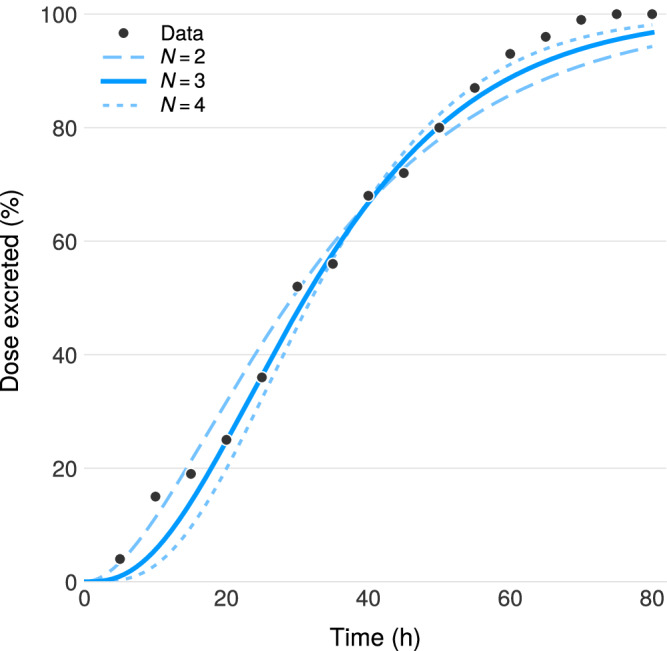
Determining colonic compartments. Colonic transit fitting. Points: radiopaque marker excretion data from Metcalf et al.^64^. Lines: model predictions for *N* = 2, 3, 4 compartments. *N* = 3 minimizes SSE.

We compared the cumulative distribution of marker excretion times to model output with only transit terms (no metabolism). We fit for *N* = 2, 3, 4 compartments, minimizing sum of squared errors (SSE) against the data. *N* = 3 provides optimal fit (Fig. 5), yielding *K*_*ct*_ = 3/35h = 0.086 h^−1^.

We next incorporate bacterial conversion of choline to TMA. Bacterial density increases along the GI tract^65^. For small intestinal compartments, we use:

26 $${b}_{i}=1{0}^{i+1}\,{\mathrm{CFU}}/{\mathrm{mL}}\,(i=1,\ldots ,7)$$

with reported values ranging from 10^*i*^ to 10^*i*+2^ CFU/mL. In the colon, bacterial density jumps substantially; we use:

27 $${b}_{{\mathrm{co}},j}=1{0}^{11.5}\,{\mathrm{CFU}}/{\mathrm{mL}}\,(j=1,2,3)$$

with reported values ranging from 10^11^ to 10^12^ CFU/mL, reflecting the substantially higher bacterial density in the colon where the majority of TMA production occurs.

Bacterial conversion of choline to TMA follows Michaelis-Menten kinetics:

28 $$g(b,c)\equiv \frac{{{\mathcal{V}}}_{{\mathrm{max}}}\,b\,c}{{K}_{{\mathrm{mb}}}+c}$$

where ${{\mathcal{V}}}_{\max }$ is the maximal conversion rate per CFU and *K*_*mb*_ is the half-saturation constant. Parameters were extracted from Day-Walsh et al.^66^ (Table 1).

**Table 1:** Microbiome model parameters

Parameter	Description	Value	Unit	Source
*K*_*e*_	Gastric emptying rate	0.984	h^−1^	^79^
*K*_*t*_	Small intestinal transit rate	2.069	h^−1^	^33^
*K*_*ct*_	Colonic transit rate	0.086	h^−1^	^64^
*K*_*ac*_	Choline absorption rate (SI)	0.14	h^−1^	^80^
*K*_*ac*,co_	Choline absorption rate (colon)	0	h^−1^	This work
*K*_*aφ*_	TMA absorption rate (SI)	0.744	h^−1^	^81^
*K*_*aφ*,co_	TMA absorption rate (colon)	0.372	h^−1^	This work
${{\mathcal{V}}}_{\max }$	Bacterial ${V}_{\max }$	130	fM h^−1^ (CFU/mL)^−1^	^66^
*K*_*mb*_	Bacterial *K*_*m*_	151	μM	^66^

Colonic choline absorption is substantially lower than in the small intestine; we assume *K*_*ac*,co_ ∈ [0, 0.2] × *K*_*ac*_, reflecting the colon’s primary role in water and electrolyte rather than nutrient absorption. For the simulations presented here, we set *K*_*ac*,co_ = 0. Colonic compartment fluid volumes of 50, 30, and 20 mL were used for the ascending, transverse, and descending colon, respectively.

Let *c*_*i*_(*t*) and *φ*_*i*_(*t*) denote choline and TMA concentrations (mmol) in small intestinal compartment *i*, with *c*_co,*j*_(*t*) and *φ*_co,*j*_(*t*) denoting the analogous colonic concentrations. Subscripts ‘s’ and ‘pl’ denote stomach and plasma. The full system is presented in Table 2.

**Table 2:** Microbiome model: ordinary differential equations for choline and TMA dynamics

Choline (*c*)
$$\frac{d{c}_{s}}{dt}=-{K}_{e}{c}_{s}$$
$$\frac{d{c}_{1}}{dt}={K}_{e}{c}_{s}-{K}_{t}{c}_{1}-{K}_{ac}{c}_{1}-g({b}_{1},{c}_{1})$$
$$\frac{d{c}_{i}}{dt}={K}_{t}({c}_{i-1}-{c}_{i})-{K}_{ac}{c}_{i}-g({b}_{i},{c}_{i})\,(i=2,\ldots ,7)$$
$$\frac{d{c}_{co,1}}{dt}={K}_{t}{c}_{7}-{K}_{ct}{c}_{co,1}-g({b}_{co,1},{c}_{co,1})$$
$$\frac{d{c}_{co,j}}{dt}={K}_{ct}({c}_{co,j-1}-{c}_{co,j})-g({b}_{co,j},{c}_{co,j})\,(j=2,3)$$
$$\frac{d{c}_{pl}}{dt}={K}_{ac}{\sum }_{i=1}^{7}{c}_{i}$$
**TMA**(***φ***)
$$\frac{d{\varphi }_{1}}{dt}=g({b}_{1},{c}_{1})-{K}_{t}{\varphi }_{1}-{K}_{a\varphi }{\varphi }_{1}$$
$$\frac{d{\varphi }_{i}}{dt}={K}_{t}({\varphi }_{i-1}-{\varphi }_{i})+g({b}_{i},{c}_{i})-{K}_{a\varphi }{\varphi }_{i}\,(i=2,\ldots ,7)$$
$$\frac{d{\varphi }_{co,1}}{dt}={K}_{t}{\varphi }_{7}-{K}_{ct}{\varphi }_{co,1}+g({b}_{co,1},{c}_{co,1})-{K}_{a\varphi,co }{\varphi }_{co,1}$$
$$\frac{d{\varphi }_{co,j}}{dt}={K}_{ct}({\varphi }_{co,j-1}-{\varphi }_{co,j})+g({b}_{co,j},{c}_{co,j})-{K}_{a\varphi,co }{\varphi }_{co,j}\,(j=2,3)$$
$$\frac{d{\varphi }_{pl}}{dt}={K}_{a\varphi }({\sum }_{i=1}^{7}{\varphi }_{i}+{\sum }_{j=1}^{3}{\varphi }_{co,j})$$

#### Native microbiome validation

To validate the model’s representation of gut bacterial metabolism, we compiled urinary TMA and TMAO excretion data from three independent human studies spanning five decades. De la Huerga and Popper (1951) administered 2–8 g choline base (15–60 mmol) to healthy subjects and measured total urinary trimethylamines (TTMA) via Reinecke salt precipitation, reporting 60–67% dose recovery within 24 h with 95–97% appearing as TMAO^67^. Zeisel et al. administered 27 mmol choline chloride to healthy volunteers (*n* = 6) and measured urinary TMA excretion of approximately 17.5 mmol/24 h (65% recovery)^68^. Zhang et al. administered 15 mmol choline chloride to six healthy males and quantified combined TMA and TMAO excretion using gas chromatography with titanous chloride reduction, reporting 62.9 ± 13.1% dose recovery^69^. The remarkable consistency of the ~63–65% bacterial conversion fraction across independent studies using different analytical methods provides robust validation targets.

**Fig. 6:**
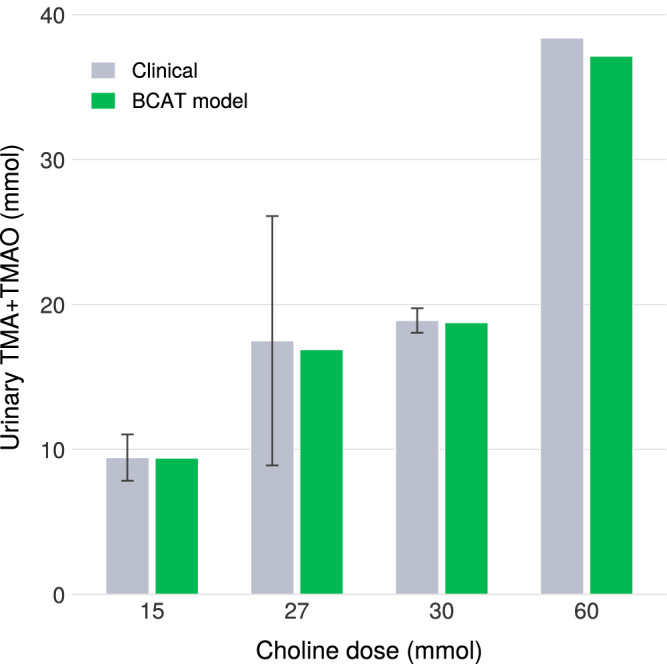
Validating BCAT output. Validation of BCAT against human urinary TMA/TMAO data. Model predictions compared to De la Huerga & Popper^67^, Zeisel et al.^68^, and Zhang et al.^69^ across doses of 15–60 mmol.

We simulated each clinical dosing protocol using the BCAT framework, which incorporates compartmental GI transit, saturable choline absorption in the small intestine, and bacterial TMA production in the colon. The model predicted TTMA excretion within the observed ranges across all four dose levels (15, 27, 30, and 60 mmol), confirming that the bacterial kinetic parameters accurately capture native microbiome choline metabolism independent of dose (Fig. 6).

### Extending BCAT to live biotherapeutics

Having validated the model’s representation of native microbiome metabolism, we now introduce the therapeutic layer: an orally administered LBP engineered to degrade a harmful intermediate in the gut lumen before systemic absorption.

#### Probiotic transit and enzymatic degradation

We model enzymatic degradation by the probiotic using Michaelis-Menten kinetics:

29 $$f(p,\varphi )\equiv \frac{{k}_{cat}\,{E}_{eq}\,p\,\varphi }{{K}_{m}+\varphi }$$

where *k*_cat_ is the catalytic rate constant, *E*_eq_ is the equilibrium enzyme concentration per cell, *p* is the local probiotic density, *φ* is the target substrate concentration, and *K*_*m*_ is the half-saturation constant. The product ${V}_{\max }={k}_{cat}\cdot {E}_{eq}$ represents the maximum reaction velocity per cell.

The probiotic transits passively through the GI tract without growth or death:

30 $$\begin{array}{lll}\frac{d{p}_{{\rm{s}}}}{dt} & = & -{K}_{e}{p}_{{\rm{s}}}\\ \frac{d{p}_{1}}{dt} & = & {K}_{e}{p}_{{\rm{s}}}-{K}_{t}{p}_{1}\\ \frac{d{p}_{i}}{dt} & = & {K}_{t}({p}_{i-1}-{p}_{i})\,(i=2,\ldots ,7)\\ \frac{d{p}_{{\rm{c}}{\rm{o}},1}}{dt} & = & {K}_{t}{p}_{7}-{K}_{ct}{p}_{{\rm{c}}{\rm{o}},1}\\ \frac{d{p}_{{\rm{c}}{\rm{o}},j}}{dt} & = & {K}_{ct}({p}_{{\rm{c}}{\rm{o}},j-1}-{p}_{{\rm{c}}{\rm{o}},j})\,(j=2,3)\end{array}$$

The substrate equations (Table 2) gain a degradation term − *f*(*p*_*k*_, *φ*_*k*_) in each compartment, representing probiotic-mediated removal of the harmful intermediate. All state variables are initialized to zero except *c*_s_(0) = *c*_0_ (dietary substrate dose) and *p*_s_(0) = *p*_0_ (administered probiotic dose). Unless otherwise stated, the probiotic is co-administered with the meal. For enzymatic parameters, see Table 3.

**Table 3:** Kinetic parameters for trimethylamine monooxygenase (TMM)

Parameter	Description	Value	Unit	Source
*K*_*m*_	Michaelis constant (TMA)	21.6 ± 1.9	μM	^55^
*k*_cat_	Maximum reaction velocity	1133.6 ± 58.6	nmol ⋅ min^−1^ ⋅ mg^−1^	^55^
*E*_eq_	Enzyme per cell	1.0 × 10^−9^	mg/CFU	^82^

#### Validation: Phenylketonuria (SYNB1618)

To validate the BCAT framework’s generalizability, we applied the model to SYNB1618, an engineered *E. coli* Nissle strain expressing phenylalanine ammonia lyase (PAL) for phenylketonuria (PKU) treatment^40^. This system provides clinical data against which to test the framework before applying it to TMAU, where no probiotic clinical trials exist.

SYNB1618 degrades phenylalanine (Phe) via the pathway:

$${\mathrm{Phe}}\mathop{{\to }}\limits^{{\rm{PAL}}}{\mathrm{TCA}}\mathop{{\to }}\limits^{{\rm{Hepatic}}}{\mathrm{HA}}$$

where TCA is trans-cinnamic acid and HA is hippuric acid. The probiotic intercepts Phe in the gut lumen before absorption, converting it to TCA, which is subsequently absorbed, metabolized to HA in the liver, and excreted in urine.

Enzyme kinetics were derived from Charbonneau et al.’s in vitro characterization (Table 4). Bacterial CFU transit dynamically through compartments rather than assuming static distribution.

**Table 4:** BCAT parameters for SYNB1618 (PKU) validation

Parameter	Description	Value	Unit	Source
*k*_*a*,Phe_	Phe absorption rate (SI)	2.88	h^−1^	^42^
*k*_*a*,TCA_	TCA absorption rate (SI)	2.7	h^−1^	^42^
*k*_*a*,TCA,colon_	TCA absorption rate (colon)	1.35	h^−1^	—
*β*	Phe absorption efficiency	[dynamic]	—	^83^
*K*_*m*_	Michaelis constant (Phe)	18.4	μM	^40^
${V}_{\max }$	Max reaction velocity	0.972	fmol ⋅ h^−1^ ⋅ CFU^−1^	^40^

*β* computed dynamically as *J*_BL_/*J*_AP_ per the carrier-mediated transport model of Hu & Borchardt (1992). Colon TCA absorption set to 50% of SI rate; colon Phe absorption assumed negligible.

Clinical data were extracted from Charbonneau et al., who reported 6-h cumulative urinary HA at four dose levels (1 × 10^10^, 5 × 10^10^, 7 × 10^10^, and 1 × 10^11^ CFU) in healthy volunteers administered a standardized phenylalanine meal.

**Fig. 7:**
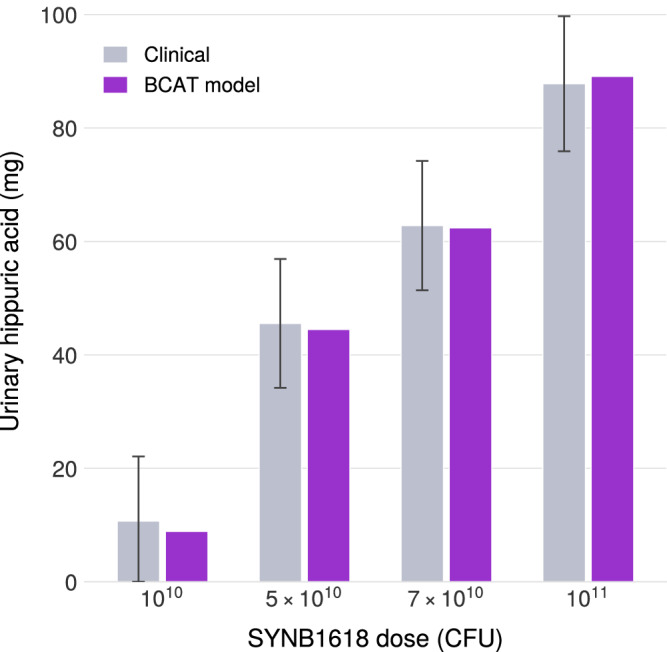
Validating LBP terms of BCAT. Validation of BCAT framework against SYNB1618 clinical data. Model predictions compared to observed 6-h cumulative urinary hippuric acid from Charbonneau et al.^40^ across four dose levels (1 × 10^10^ to 1 × 10^11^ CFU). Mean prediction error: 5%.

The BCAT framework achieved mean prediction error of 5% across all dose levels, compared to approximately 30% error reported by Charbonneau et al. using their two-compartment model (Fig. 7). The improved accuracy is attributable to two structural advances:

1. Extension to 10 compartments (7 SI + 3 colon), capturing transit-dependent substrate-bacteria exposure more accurately than lumped models (see Tables 5 and 6).
2. Explicit modeling of bidirectional absorption dynamics with an *β*-factor adapted from Mays and Nair^42^, a quasi-steady-state correction accounting for enteric recirculation and active transport.

**Table 5:** SYNB1618 model: ordinary differential equations for Phe and TCA dynamics

Phenylalanine (*φ*)
$$\frac{d{\varphi }_{s}}{dt}=-{K}_{e}{\varphi }_{s}-f({p}_{s},{\varphi }_{s})$$
$$\frac{d{\varphi }_{1}}{dt}={K}_{e}{\varphi }_{s}-{K}_{t}{\varphi }_{1}-\beta {k}_{a,\varphi }{\varphi }_{1}-f({p}_{1},{\varphi }_{1})$$
$$\frac{d{\varphi }_{i}}{dt}={K}_{t}({\varphi }_{i-1}-{\varphi }_{i})-\beta {k}_{a,\varphi }{\varphi }_{i}-f({p}_{i},{\varphi }_{i})\,(i=2,\ldots ,7)$$
$$\frac{d{\varphi }_{co,1}}{dt}={K}_{t}{\varphi }_{7}-{K}_{ct}{\varphi }_{co,1}-f({p}_{co,1},{\varphi }_{co,1})$$
$$\frac{d{\varphi }_{co,j}}{dt}={K}_{ct}({\varphi }_{co,j-1}-{\varphi }_{co,j})-f({p}_{co,j},{\varphi }_{co,j})\,(j=2,3)$$
**Trans-cinnamic acid**(*τ*)
$$\frac{d{\tau }_{s}}{dt}=f({p}_{s},{\varphi }_{s})-{K}_{e}{\tau }_{s}$$
$$\frac{d{\tau }_{1}}{dt}={K}_{e}{\tau }_{s}+f({p}_{1},{\varphi }_{1})-{K}_{t}{\tau }_{1}-{k}_{a,\tau }{\tau }_{1}$$
$$\frac{d{\tau }_{i}}{dt}={K}_{t}({\tau }_{i-1}-{\tau }_{i})+f({p}_{i},{\varphi }_{i})-{k}_{a,\tau }{\tau }_{i}\,(i=2,\ldots ,7)$$
$$\frac{d{\tau }_{co,1}}{dt}={K}_{t}{\tau }_{7}+f({p}_{co,1},{\varphi }_{co,1})-{K}_{ct}{\tau }_{co,1}-{k}_{a,\tau }^{co}{\tau }_{co,1}$$
$$\frac{d{\tau }_{co,j}}{dt}={K}_{ct}({\tau }_{co,j-1}-{\tau }_{co,j})+f({p}_{co,j},{\varphi }_{co,j})-{k}_{a,\tau }^{co}{\tau }_{co,j}\,(j=2,3)$$
$$\frac{dHA}{dt}={k}_{a,\tau }{\sum }_{i=1}^{7}{\tau }_{i}+{k}_{a,\tau }^{co}{\sum }_{j=1}^{3}{\tau }_{co,j}$$

**Table 6:** BCAT model with LBP intervention: ordinary differential equations

TMA (*φ*)
$$\frac{d{\varphi }_{1}}{dt}=g({b}_{1},{c}_{1})-f({p}_{1},{\varphi }_{1})-{K}_{t}{\varphi }_{1}-{K}_{a\varphi }{\varphi }_{1}$$
$$\frac{d{\varphi }_{i}}{dt}={K}_{t}({\varphi }_{i-1}-{\varphi }_{i})+g({b}_{i},{c}_{i})-f({p}_{i},{\varphi }_{i})-{K}_{a\varphi }{\varphi }_{i}\,(i=2,\ldots ,7)$$
$$\frac{d{\varphi }_{co,1}}{dt}={K}_{t}{\varphi }_{7}-{K}_{ct}{\varphi }_{co,1}+g({b}_{co,1},{c}_{co,1})-f({p}_{co,1},{\varphi }_{co,1})-{K}_{a\varphi,co }{\varphi }_{co,1}$$
$$\frac{d{\varphi }_{co,j}}{dt}={K}_{ct}({\varphi }_{co,j-1}-{\varphi }_{co,j})+g({b}_{co,j},{c}_{co,j})-f({p}_{co,j},{\varphi }_{co,j})-{K}_{a\varphi,co }{\varphi }_{co,j}\,(j=2,3)$$
$$\frac{d{\varphi }_{pl}}{dt}={K}_{a\varphi }({\sum }_{i=1}^{7}{\varphi }_{i}+{\sum }_{j=1}^{3}{\varphi }_{co,j})$$

This validation demonstrates that the BCAT framework generalizes across both native microbiome metabolism (see Native Microbiome Validation) and engineered probiotic activity, supporting its utility as a platform for LBP development.

### Sobol’ sensitivity analysis

The Sobol’ decomposition partitions output variance into contributions from individual parameters and their interactions^70,71^. As long as parameters are iid, one can decompose the model output uniquely along orthogonal directions using conditional probabilities. We note that formulations of Sobol’-like sensitivities exist that do not require independence^72–74^; for the purposes of this paper, we assume parameters are independent and identically distributed (iid).

For model output *Y* = Γ^*^ and parameter *X*_*i*_, the first-order sensitivity index quantifies the main effect:

31 $${S}_{1,i}=\frac{{V}_{{X}_{i}}({{\mathbb{E}}}_{{{\bf{X}}}_{ \sim i}}(Y| {X}_{i}))}{V(Y)}$$

and the total-order index captures all effects involving *X*_*i*_, including interactions:

32 $${S}_{T,i}=\frac{{{\mathbb{E}}}_{{{\bf{X}}}_{ \sim i}}({V}_{{X}_{i}}(Y| {{\bf{X}}}_{ \sim i}))}{V(Y)}$$

where **X**_~*i*_ denotes all parameters except *X*_*i*_, *V* is variance, and ${\mathbb{E}}$ is expectation^60,63^. The difference *S*_*T*,*i*_ − *S*_1,*i*_ quantifies the contribution of interactions involving parameter *i*.

Global sensitivity indices are typically estimated using sampling from joint distributions^75^. The choice of underlying distributions can substantially affect sensitivity estimates^76,77^. Uniform or normal distributions are typical; other distributions can be used when deeper parameter knowledge is available^78^. Given the significant variation in reported gut microbiome parameters, we use uniform distributions spanning ± 50% of nominal values for most parameters, with 100-fold variation for *E*_eq_ given its dominant influence identified in Morris screening.

Indices were estimated using the Saltelli sampling scheme^61^, requiring *k* = *n*(*p* + 2) model evaluations for *p* parameters. We used *n* = 2048 samples across *p* = 10 parameters, yielding *k* = 24, 576 total evaluations. Parameter ranges are specified in Table 7.

**Table 7:** Parameter distributions for Sobol’ analysis

Parameter	Nominal	Range	Fold variation	Source
*E*_eq_	1.0 × 10^−9^	[10^−10^, 10^−8^]	100-fold	^55^
*K*_*ac*_	.14	[0.1, 2.5]	25-fold	
*k*_cat_	68.02	[34, 102]	3-fold	^55^
*K*_*t*_	2.069	[1.04, 3.10]	3-fold	^33^
*K*_*aφ*_	0.744	[0.37, 1.12]	3-fold	^81^
*f*_co_	0.5	[0.25, 0.75]	3-fold	This work
*K*_*e*_	0.984	[0.49, 1.48]	3-fold	^79^
*k*_bact_	1.0 × 10^−4^	[10^−5^, 10^−3^]	100-fold	^66^
*B*_CO_	10^11^	[10^10^, 10^13^]	1000-fold	^65^
*B*_SI_	10^2^	[10^1^, 10^4^]	1000-fold	^65^

To verify convergence, we computed *S*_1_ and *S*_*T*_ for increasing sample sizes *n* = 256, 512, 1024, 2048 and confirmed that indices stabilized to within ± 0.02 for *n* ≥ 1024.

## Data Availability

Data is available at https://www.vicenzodevito.com/signedsealeddelivered and https://github.com/V-DeVito/BCAT.

## Code availability

Code is available at https://www.vicenzodevito.com/signedsealeddelivered and https://github.com/V-DeVito/BCAT.
